# Supplementary material for: High-speed 4D neutron computed tomography for quantifying water dynamics in polymer electrolyte fuel cells
Source: Nat Commun. 2022 Mar 25;13:1616. doi: 10.1038/s41467-022-29313-5 (PMC8956593; doi:10.1038/s41467-022-29313-5)
Supplement: Supplementary file 1 — Supplementary Information [file 41467_2022_29313_MOESM1_ESM.pdf]

Supplementary Information:

High-Speed 4D Neutron Computed Tomography for Quantifying Water Dynamics in Polymer Electrolyte Fuel Cells

Ziesche et al.

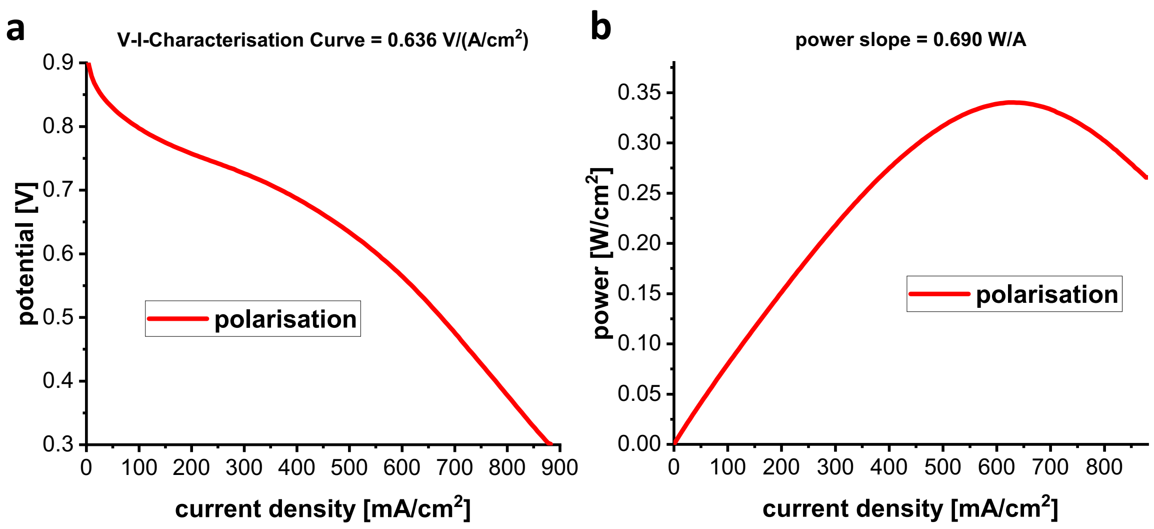

Supplementary Figure 1: Pre-beamtime laboratory testing; Polarisation and power curves of the single serpentine PEFC: a V-I characteristic with average V-I slope of 636 Ohm cm^{-2} in the working region of the cell. **b** power curve with a maximum of 340 mW cm^{-2} at a current density of 627 mA cm^{-2} and a potential of 543 mV. In the linear working region the cell exhibits shows a power slope of 690 mW A^{-1} or mV.

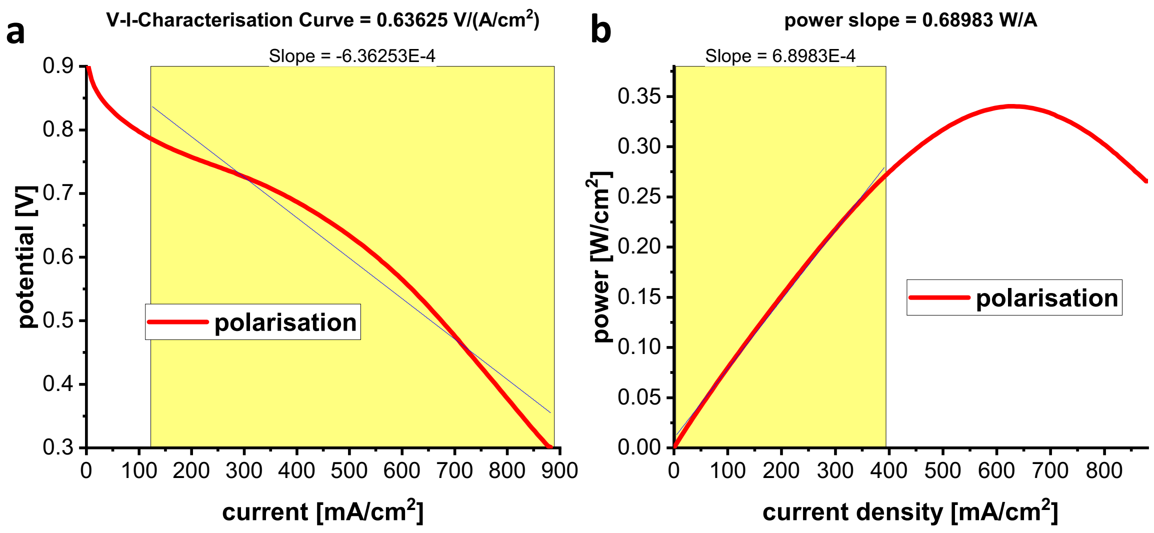

Supplementary Figure 2: Pre-beamtime laboratory testing; Polarisation and power curves of the single serpentine PEFC with fits of the V-I-characterisation and the power slope: a shows the V-I characterisation curve of the used fuel cell with a V-I slope of about 636 mV/(A/cm^2) in the working range. **b** displays the corresponding power curve with a maximum of ca. 340 mW at 543 mA and a power increase of ca. 690 mW/A in the working range.

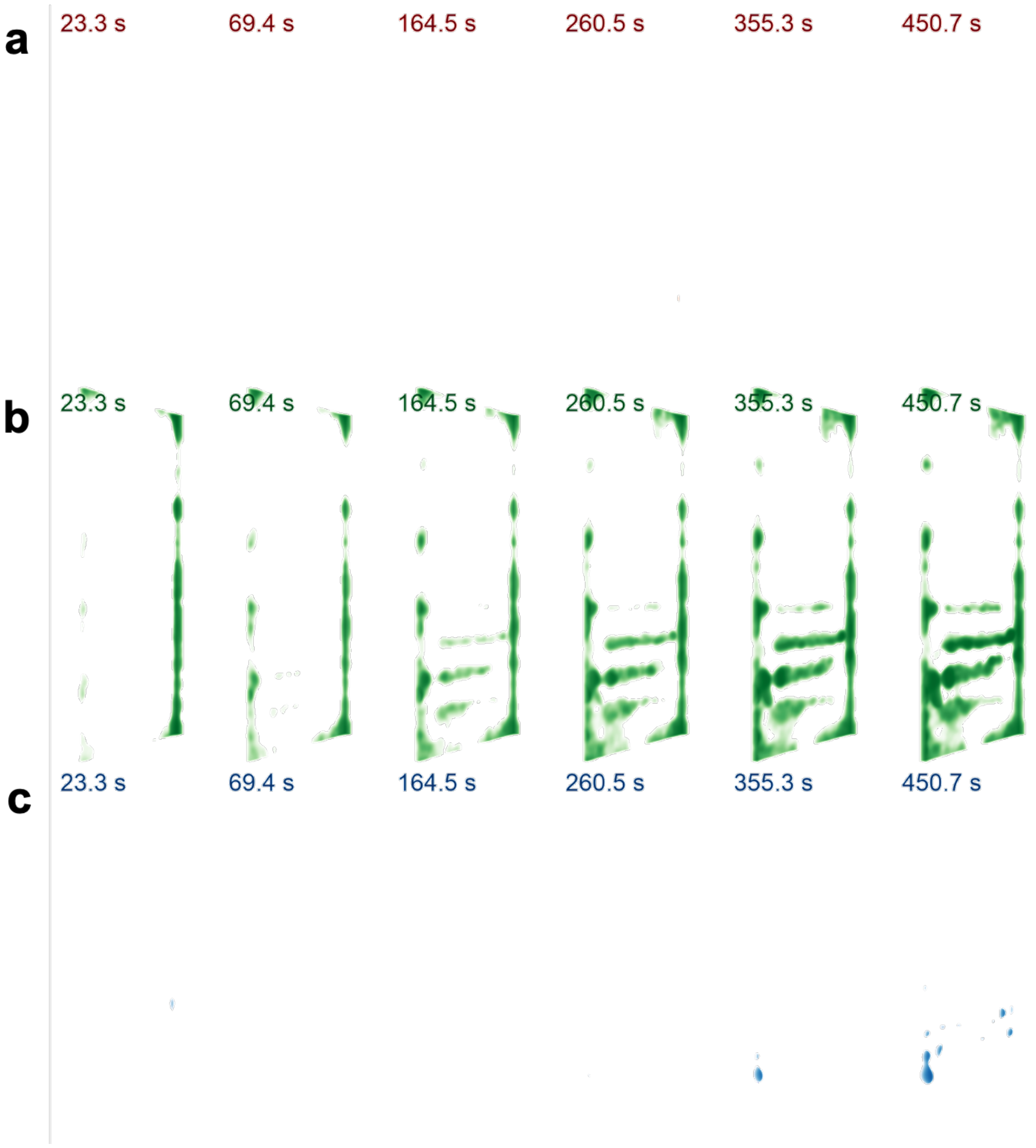

Supplementary Figure 3: 3D visualisation of the water evolution in the single serpentine PEFC. Water evolution in the **a** anode flow field (red), **b** the MEA (green) and **c** the cathode flow field (blue) at a constant current hold of 100 mA/cm^2.

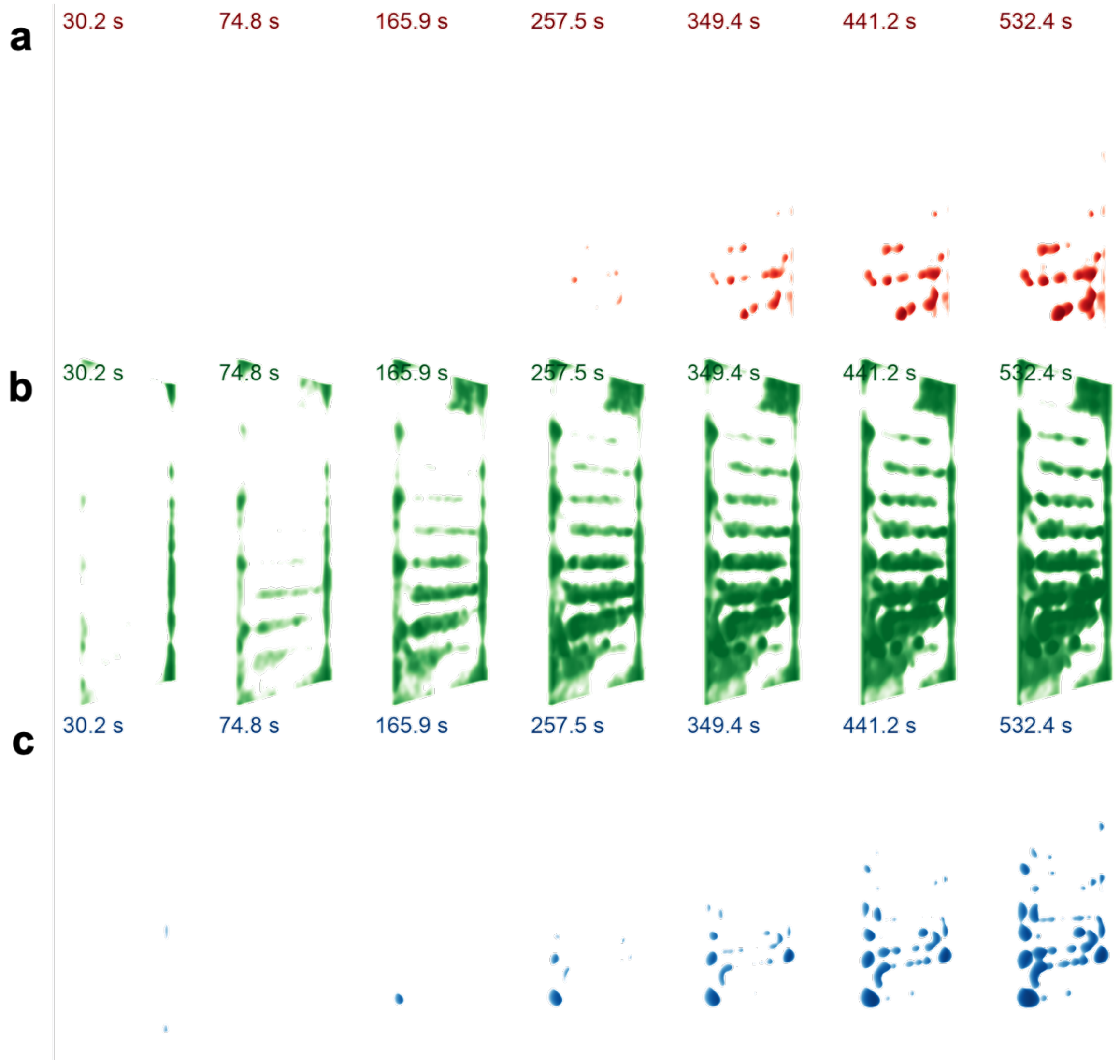

Supplementary Figure 4: 3D visualisation of the water evolution in the single serpentine PEFC. Water evolution in the **a** anode flow field (red), **b** the MEA (green) and **c** the cathode flow field (blue) at a constant current hold of 200 mA/cm^2.

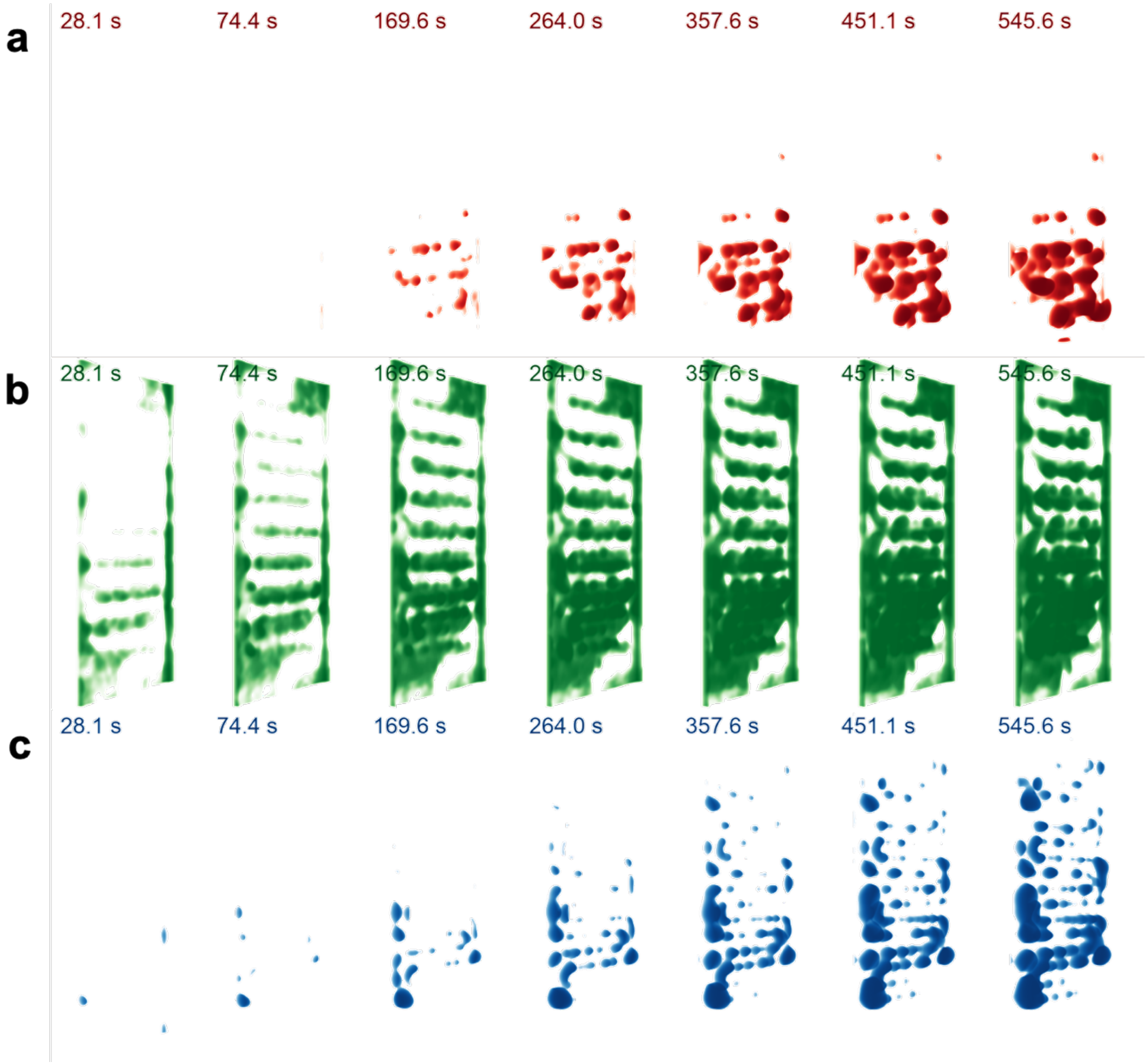

Supplementary Figure 5: 3D visualisation of the water evolution in the single serpentine PEFC. Water evolution in the **a** anode flow field (red), **b** the MEA (green) and **c** the cathode flow field (blue) at a constant current hold of 300 mA/cm^2.

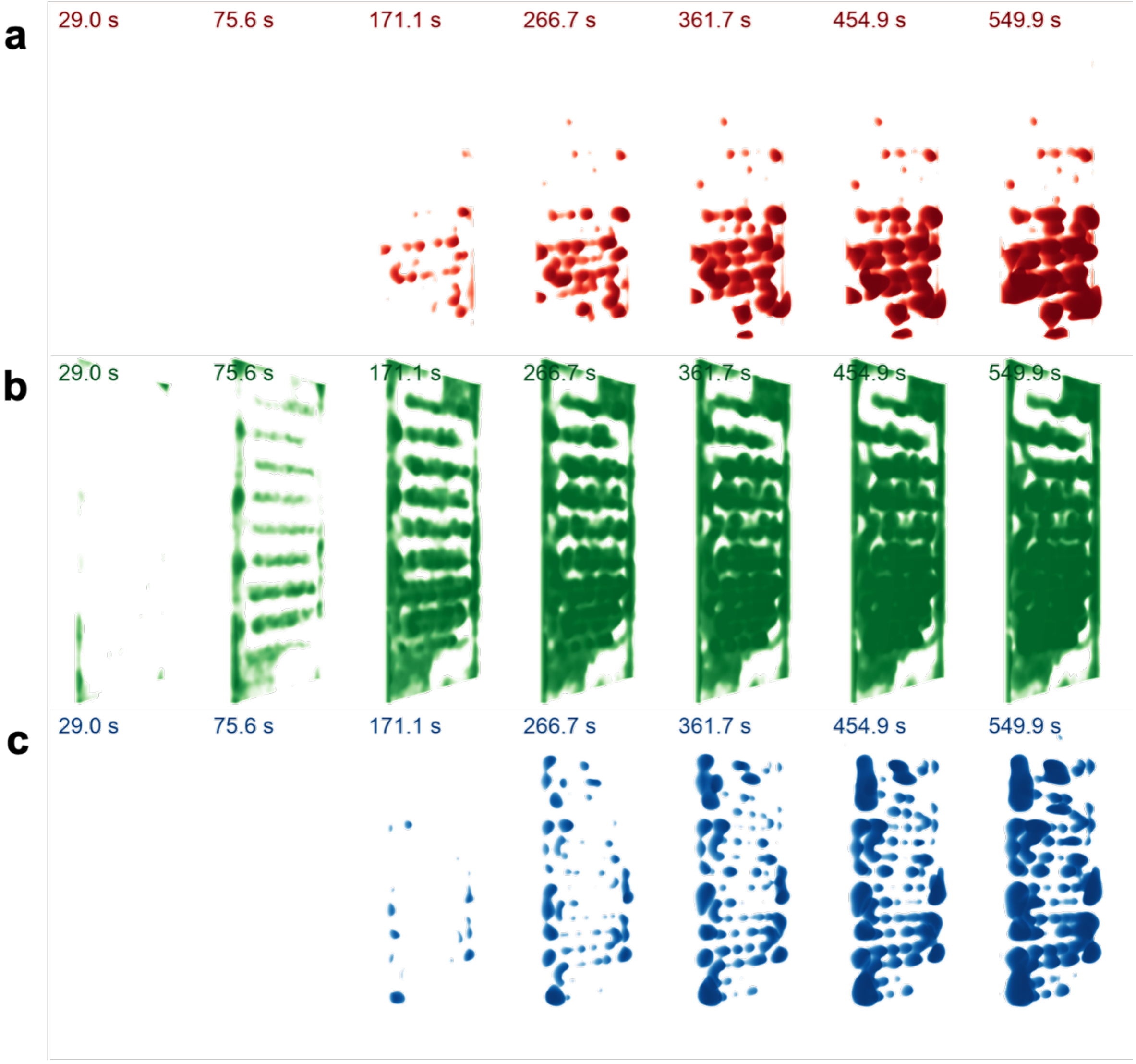

Supplementary Figure 6: 3D visualisation of the water evolution in the single serpentine PEFC. Water evolution in the **a** anode flow field (red), **b** the MEA (green) and **c** the cathode flow field (blue) at a constant current hold of 400 mA/cm^2.

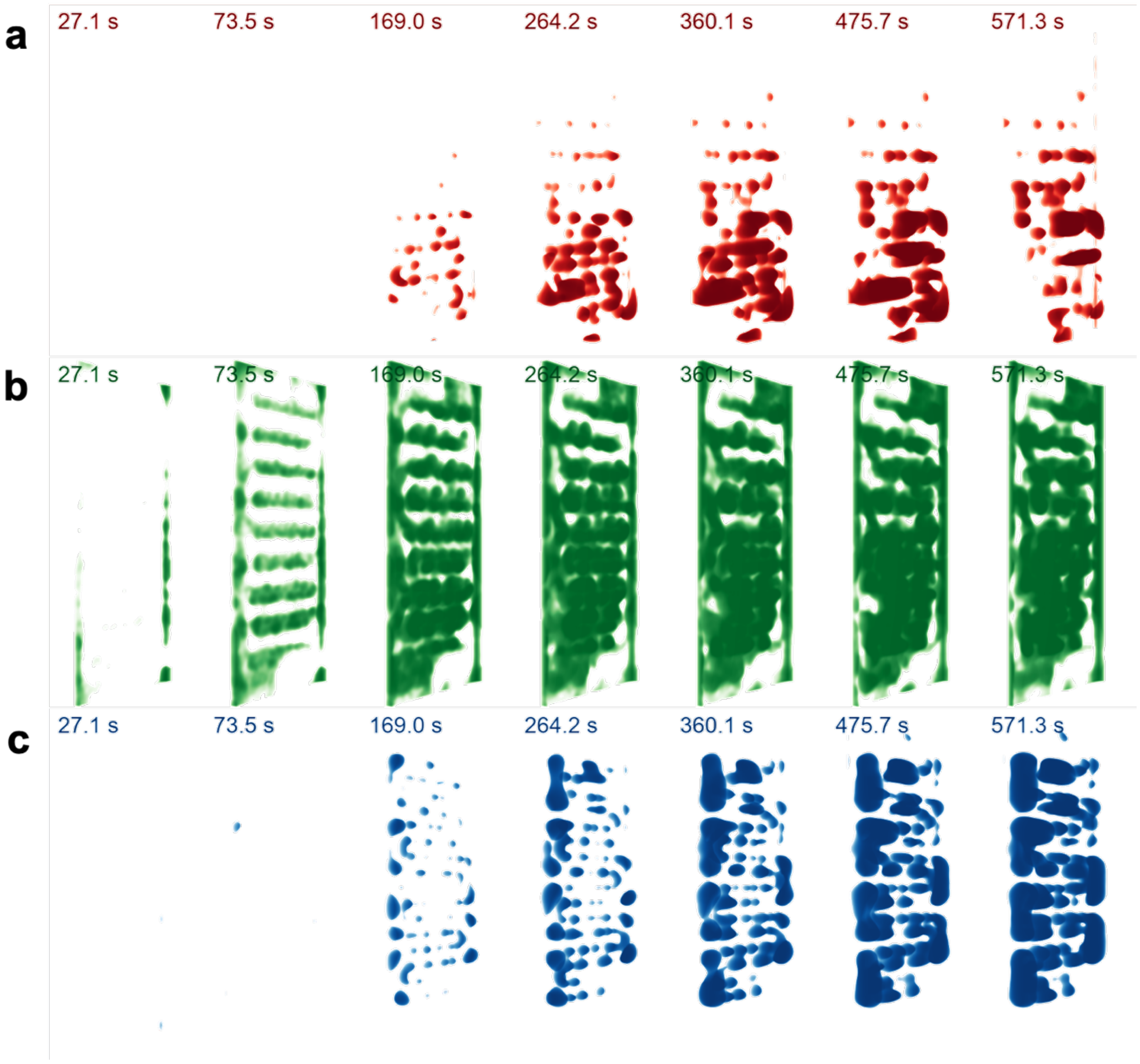

Supplementary Figure 7: 3D visualisation of the water evolution in the single serpentine PEFC. Water evolution in the **a** anode flow field (red), **b** the MEA (green) and **c** the cathode flow field (blue) at a constant current hold of 500 mA/cm^2.

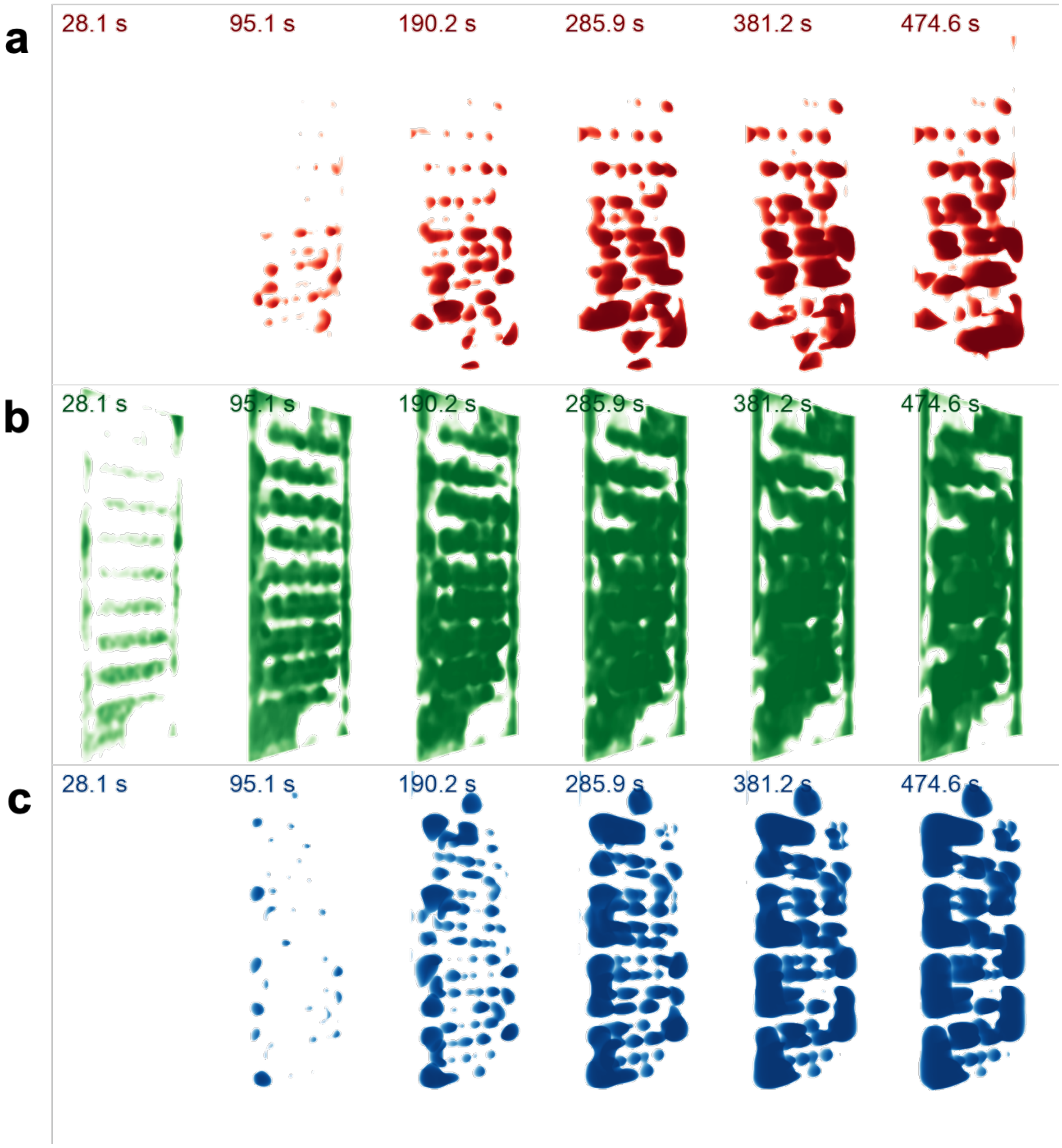

Supplementary Figure 8: 3D visualisation of the water evolution in the single serpentine PEFC. Water evolution in the **a** anode flow field (red), **b** the MEA (green) and **c** the cathode flow field (blue) at a constant current hold of 600 mA/cm^2.

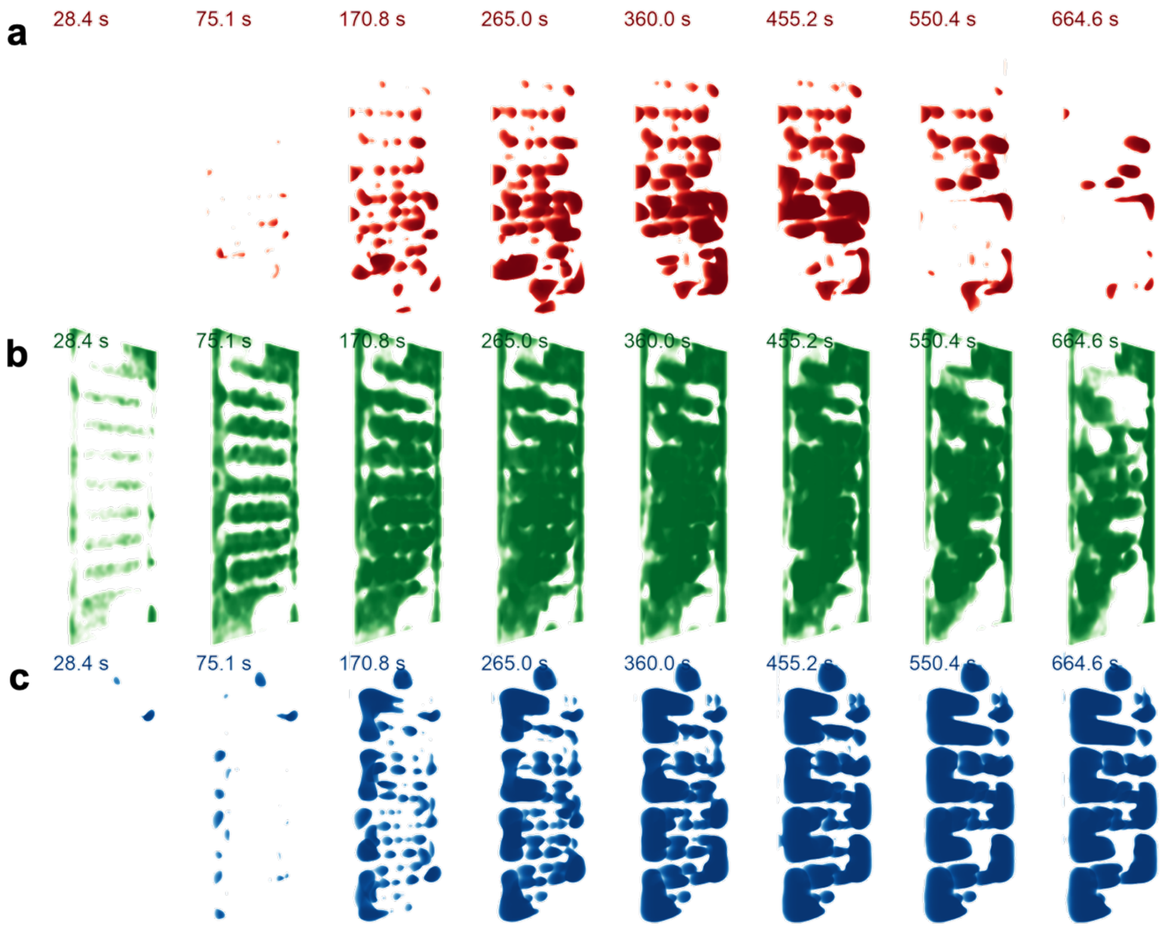

Supplementary Figure 9: 3D visualisation of the water evolution in the single serpentine PEFC. Water evolution in the **a** anode flow field (red), **b** the MEA (green) and **c** the cathode flow field (blue) at a constant current hold of 700 mA/cm^2.

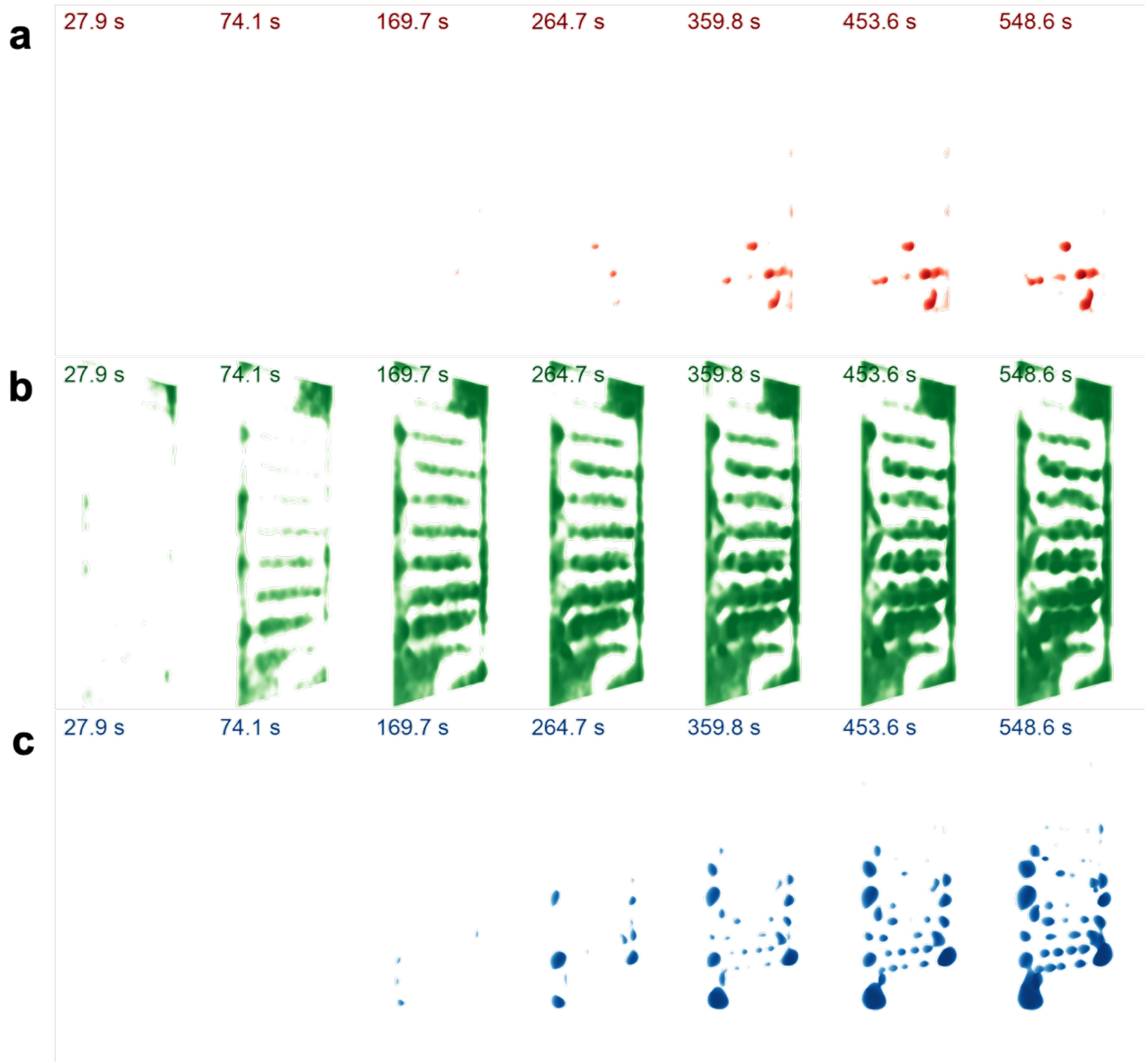

Supplementary Figure 10: 3D visualisation of the water evolution in the single serpentine PEFC. Water evolution in the **a** anode flow field (red), **b** the MEA (green) and **c** the cathode flow field (blue) at a constant potential hold at 0.7 V.

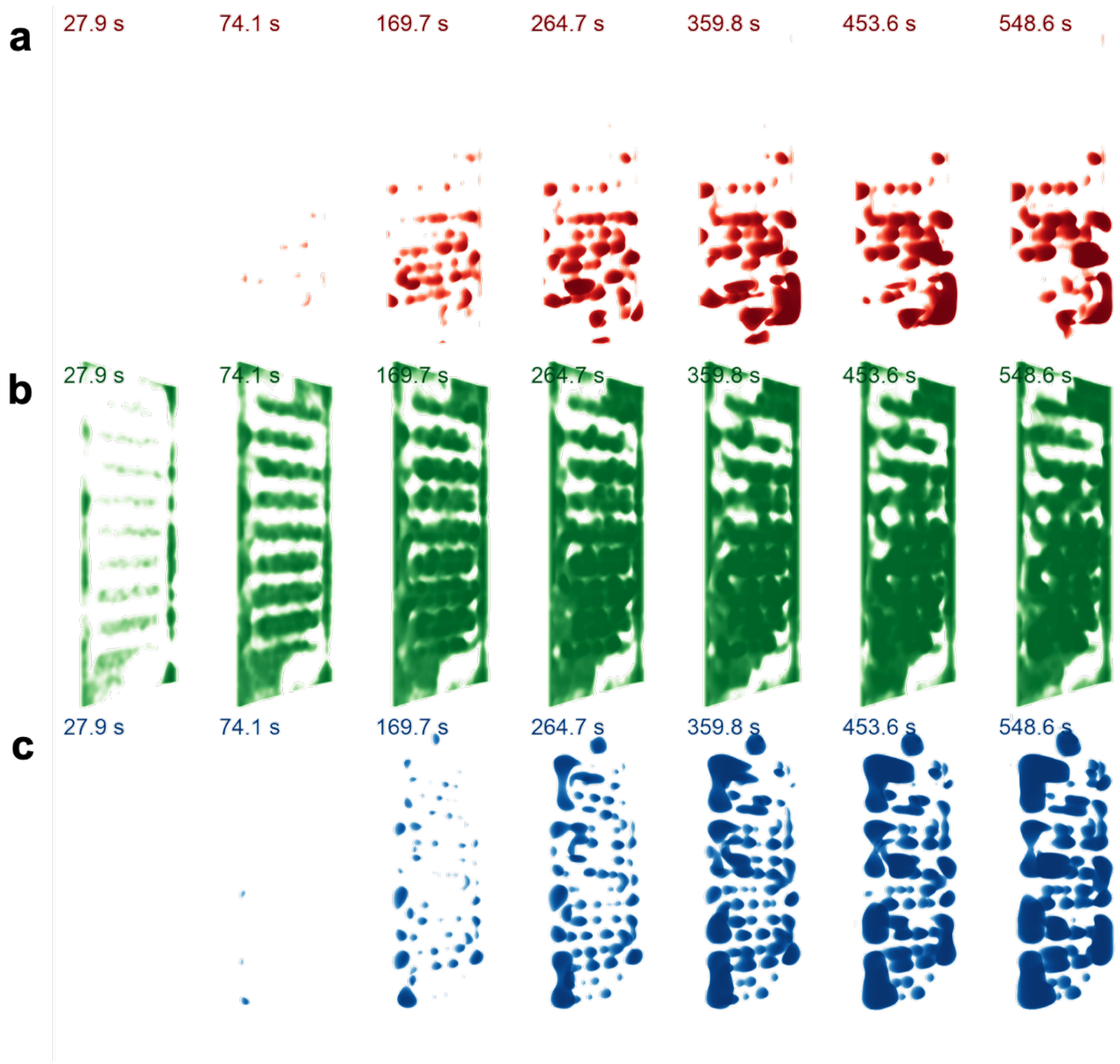

Supplementary Figure 11: 3D visualisation of the water evolution in the single serpentine PEFC. Water evolution in the **a** anode flow field (red), **b** the MEA (green) and **c** the cathode flow field (blue) at a constant potential hold at 0.5 V.

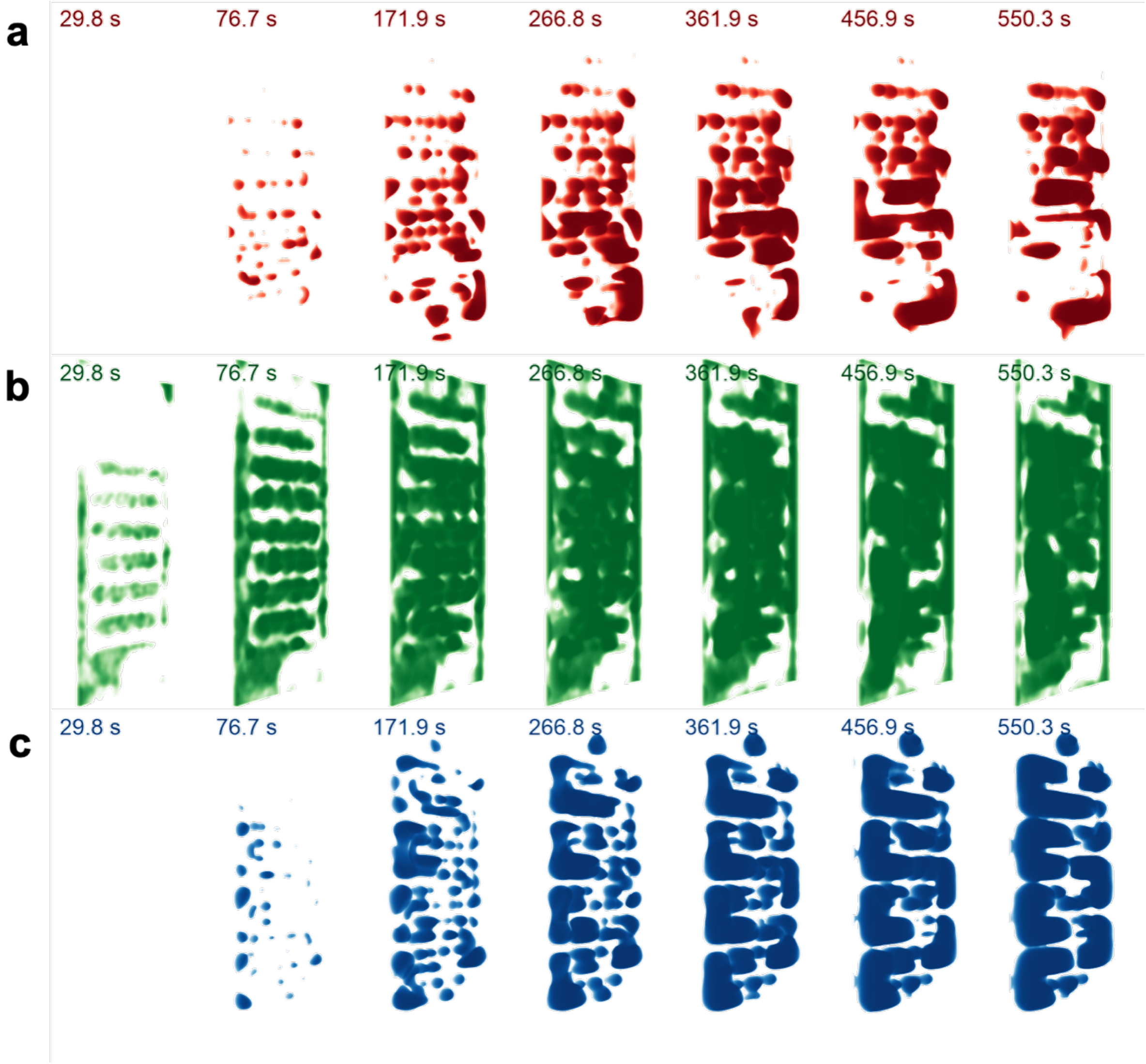

Supplementary Figure 12: 3D visualisation of the water evolution in the single serpentine PEFC. Water evolution in the **a** anode flow field (red), **b** the MEA (green) and **c** the cathode flow field (blue) at a constant potential hold at 0.3 V.

Fitting function:

$$f(t) = mt + y$$

100 mA/cm²: 0.0493 mm³/min

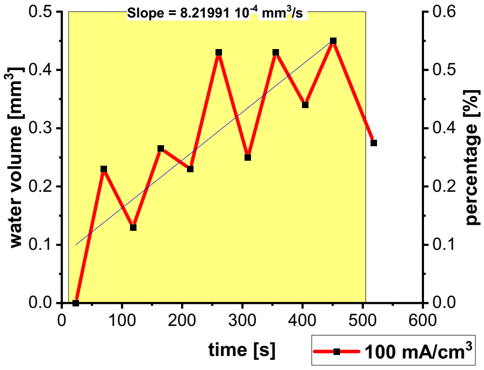

200 mA/cm²: 0.1146 mm³/min

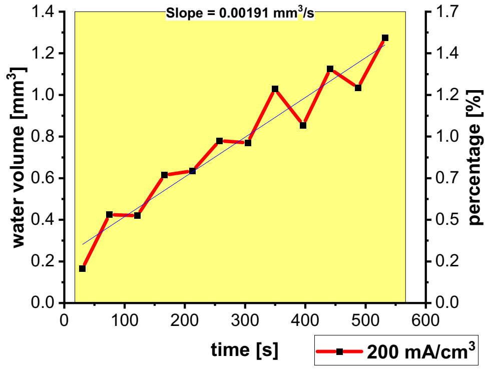

300 mA/cm²: 0.2484 mm³/min

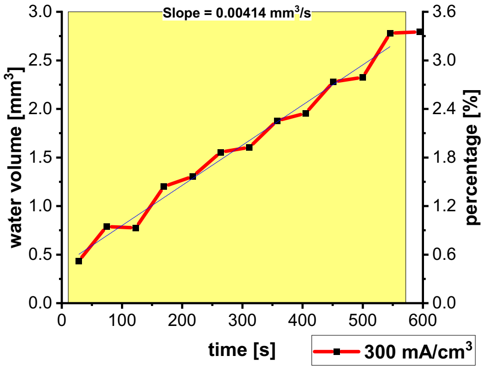

400 mA/cm²: 0.4824 mm³/min

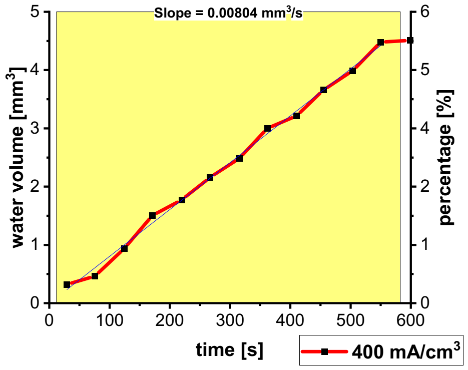

500 mA/cm²: 0.6750 mm³/min

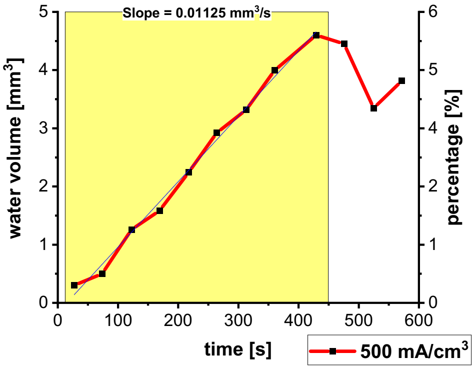

600 mA/cm²: 0.7344 mm³/min

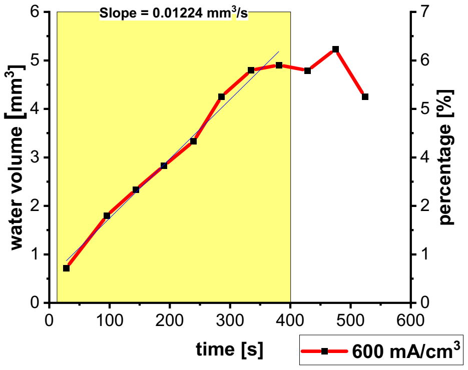

700 mA/cm^2: 0.9846 mm^3/min

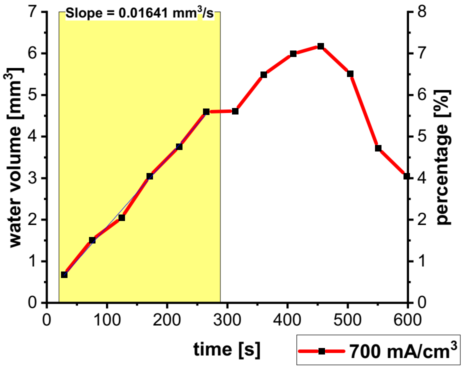

Supplementary Figure 13: Calculation of the water volume evolution inside the single serpentine anode flow field for different current holds from 100 to 700 mA/cm^2.

0.7 V: 0.0452 mm^3/min

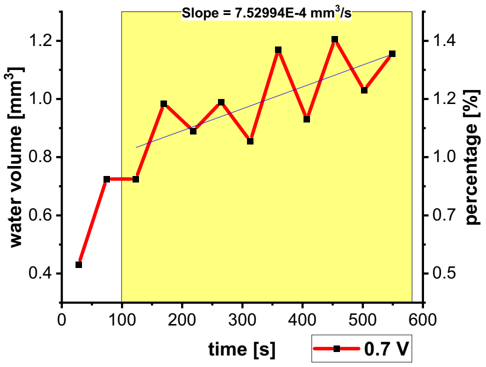

0.5 V: 0.4920 mm^3/min

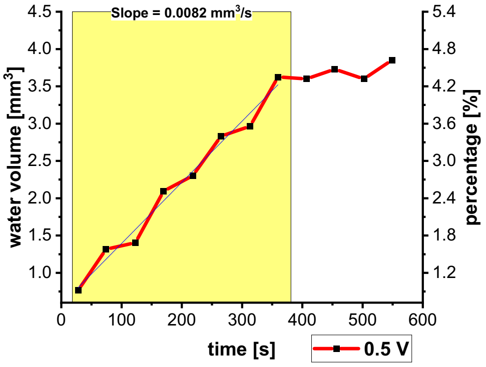

0.3 V: 1.1358 mm^3/min

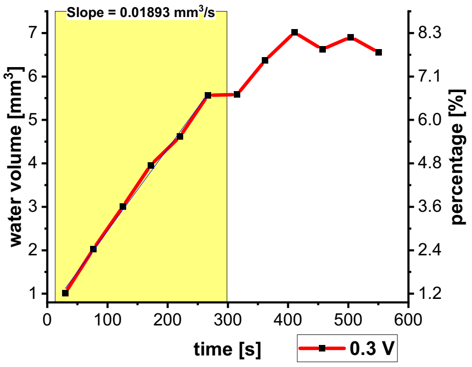

Supplementary Figure 14: Calculation of the water volume evolution inside the single serpentine anode flow field for different potential holds from 0.7 to 0.3 V.

Supplementary Table 1: Slope of the water evolution in the single serpentine anode flow field for the different current and potential holds: The determined slopes represent the time dependent water evolution in the anode flow field for different constant current densities and potentials.

Current density [mA cm^{-2}]	Water volume [mm^3 s^{-1}]	Water volume [mm^3 min^{-1}]
100	0.00082	0.0493
200	0.00191	0.1146
300	0.00414	0.2484
400	0.00804	0.4824
500	0.01125	0.6750
600	0.01224	0.7344
700	0.01641	0.9846
**Potential [V]**	**Water volume [mm^3 s^{-1}]**	**Water volume [mm^3 min^{-1}]**
0.7	0.00075	0.0452
0.5	0.0082	0.4920
0.3	0.01893	1.1358

Supplementary Table 2: Slope of the water evolution in the single serpentine cathode flow field for the different current and potential holds: The determined slopes represent the time dependent water evolution in the cathode flow field for different constant current densities and potentials.

Current density [mA cm^{-2}]	Water volume [mm^3 s^{-1}]	Water volume [mm^3 min^{-1}]
100	0.00154	0.0924
200	0.00378	0.2268
300	0.00761	0.4566
400	0.01152	0.6912
500	0.01601	0.9606
600	0.02256	1.3536
700	0.0266	1.5960
**Potential [V]**	**Water volume [mm^3 s^{-1}]**	**Water volume [mm^3 min^{-1}]**
0.7	0.00494	0.2964
0.5	0.0151	0.9060
0.3	0.02912	1.7472

Fitting function:

$$f(t) = mt + y$$

100 mA/cm^2: 0.0924 mm^3/min

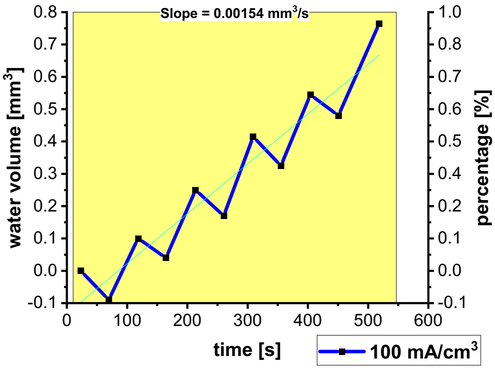

200 mA/cm^2: 0.2268 mm^3/min

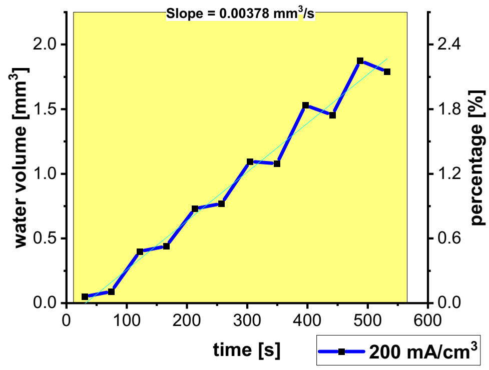

300 mA/cm^2: 0.4566 mm^3/min

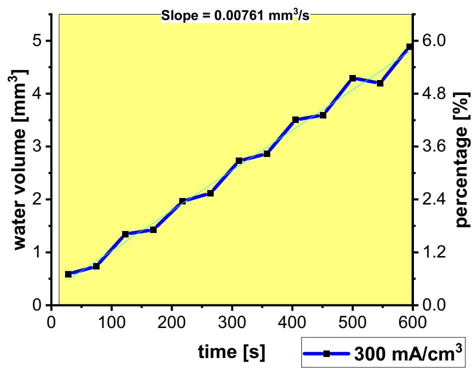

400 mA/cm^2: 0.6912 mm^3/min

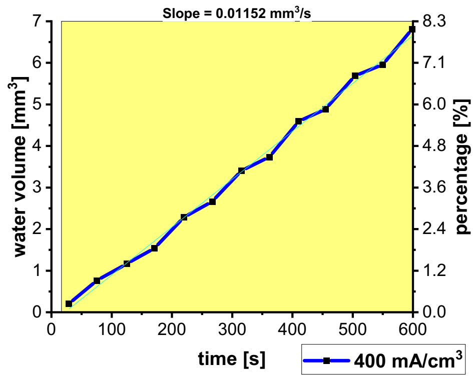

500 mA/cm^2: 0.9606 mm^3/min

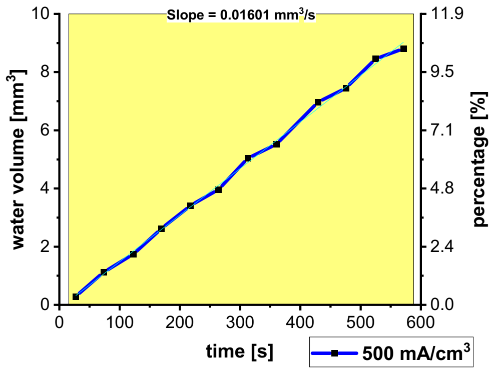

600 mA/cm^2: 1.3536 mm^3/min

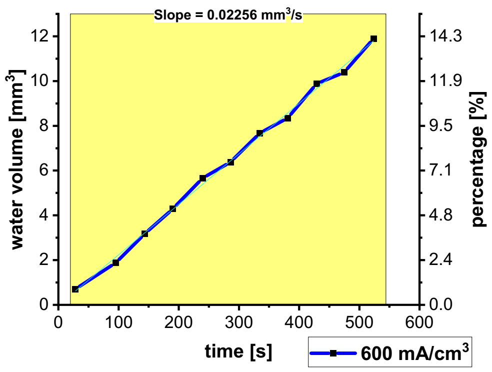

700 mA/cm^2: 1.5960 mm^3/min

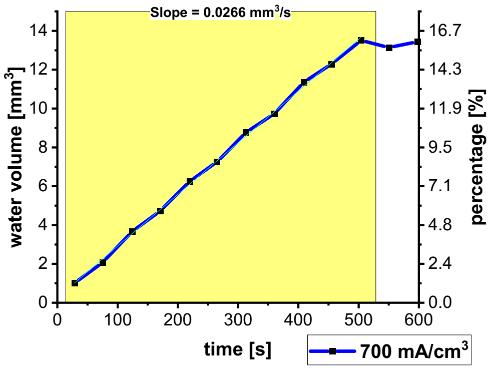

Supplementary Figure 15: Calculation of the water volume evolution inside the single serpentine cathode flow field for different current holds from 100 to 700 mA/cm^2.

0.7 V: 0.2964 mm^3/min

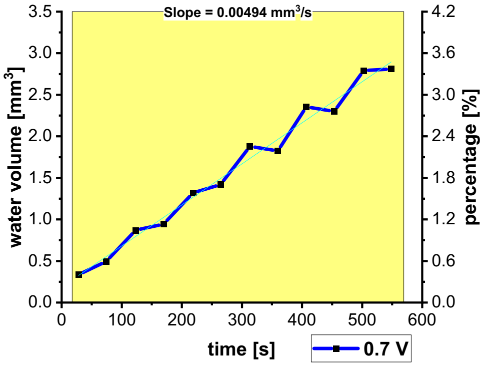

0.5 V: 0.9060 mm^3/min

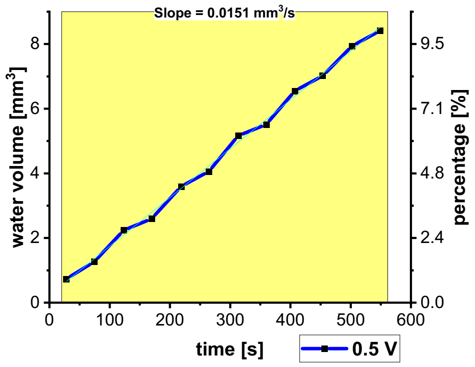

0.3 V: 1.7472 mm^3/min

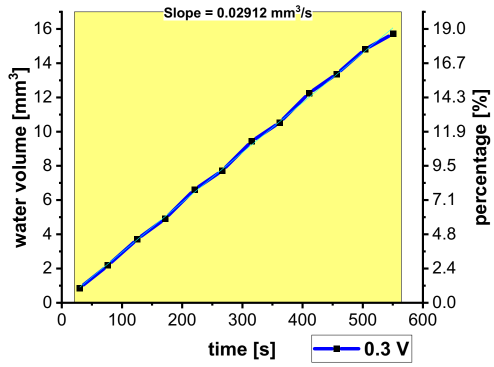

Supplementary Figure 16: Calculation of the water volume evolution inside the single serpentine cathode flow field for different potential holds from 0.7 to 0.3 V.

Fitting function:

$$f(t) = a\left(1 - e^{-\frac{t}{\tau}}\right) + b$$

100 mA/cm²: 0.75 mm³

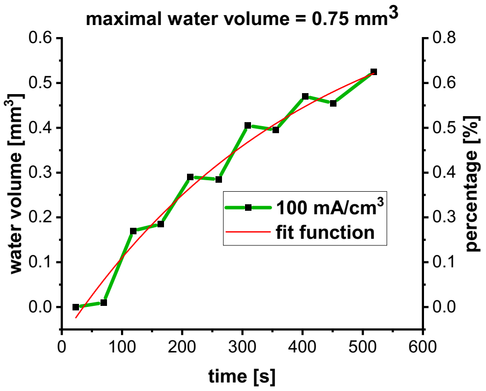

200 mA/cm²: 1.46 mm³

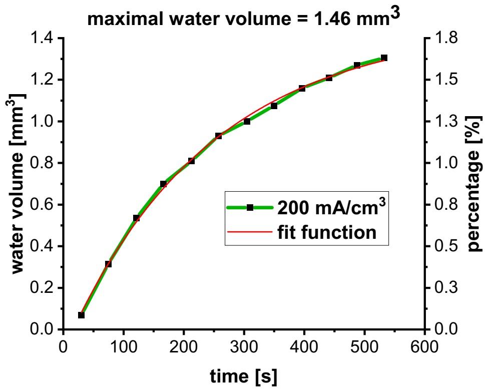

300 mA/cm²: 2.15 mm³

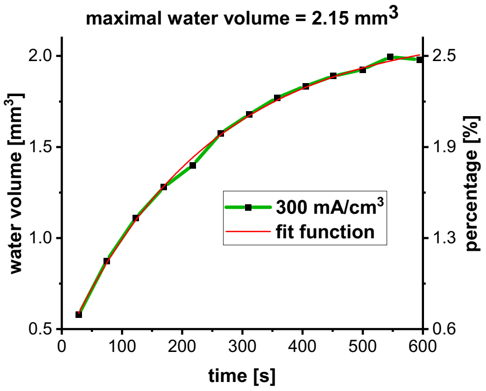

400 mA/cm²: 2.33 mm³

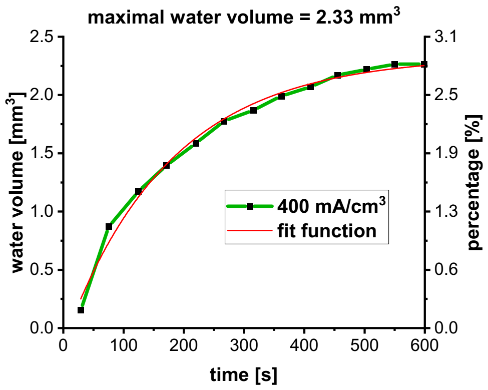

500 mA/cm²: 2.23 mm³

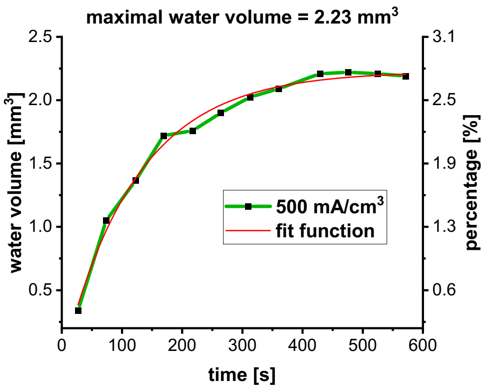

600 mA/cm²: 2.47 mm³

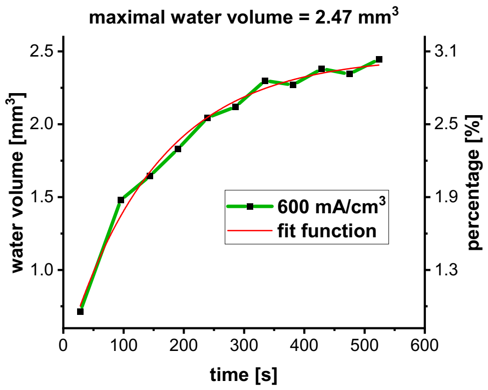

700 mA/cm^2: 2.43 mm^3

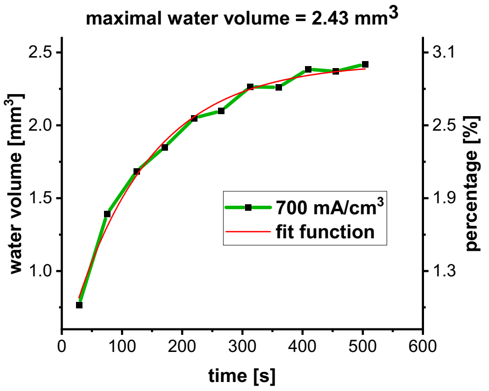

Supplementary Figure 17: Calculation of the maximal water saturation volume evolution inside the MEA of the single serpentine flow field fuel cell for different current holds from 100 to 700 mA/cm^2.

0.7 V: 1.31 mm^3

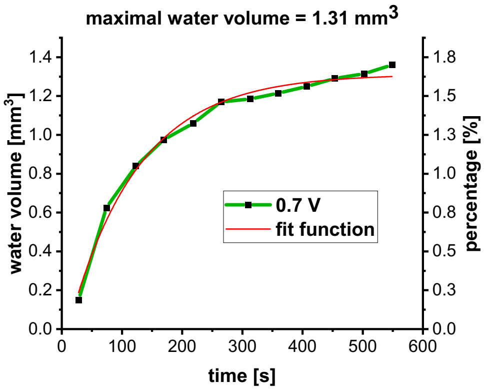

0.5 V: 2.05 mm^3

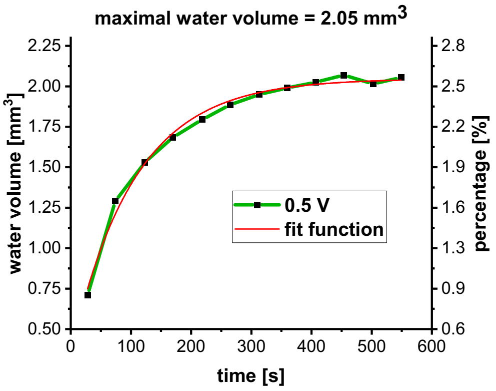

0.3 V: 2.53 mm^3

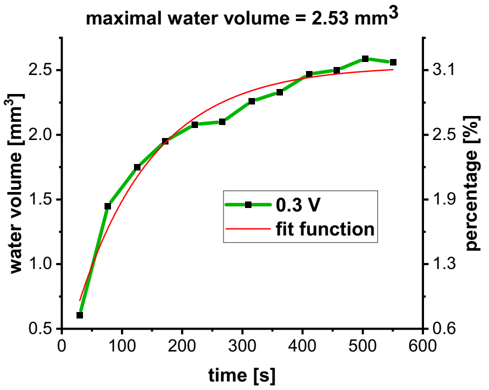

Supplementary Figure 18: Calculation of the maximal water saturation volume evolution inside the MEA of the single serpentine flow field fuel cell for different potential holds from 0.7 to 0.3 V.

Supplementary Table 3: Current and potential dependent maximal water volume evolution in the MEA: The maximal water volumes in the MEA show a current density and potential dependent behaviour and seem to converge towards a constant value for higher powers.

Current density [mA cm^{-2}]	Maximal water volume [mm^3]
100	0.75
200	1.46
300	2.15
400	2.33
500	2.23
600	2.47
700	2.43
**Potential [V]**	**Maximal water volume [mm^3]**
0.7	1.31
0.5	2.05
0.3	2.53

Supplementary Table 4: Calculated times to reach 95 % and 99 % of the maximal water volume in the MEA and the equilibrium state for different current densities and potentials.

Current density [mA cm^{-2}]	$t_{equilibrium}$ (p = 0.95) [s]	$t_{equilibrium}$ (p = 0.99) [s]
100	1283	2183
200	724	1061
300	663	1021
400	530	787
500	373	581
600	424	662
700	366	579
**Potential [V]**	**$t_{equilibrium}$ (p = 0.95) [s]**	**$t_{equilibrium}$ (p = 0.99) [s]**
0.7	369	634
0.5	298	479
0.3	365	554

Supplementary Table 5: Experimentally determined water volume slope of the current and potential hold measurements.

Current density [mA cm^{-2}]	Water slope [mm^3 s^{-1}]	Water slope [mm^3 min^{-1}]
100	0.00323	0.1938
200	0.00798	0.4788
300	0.01406	0.8436
400	0.02252	1.3512
500	0.03159	1.8954
600	0.04087	2.4522
700	0.04475	2.6850
**Potential [V]**	**Water slope [mm^3 s^{-1}]**	**Water slope [mm^3 min^{-1}]**
0.7	0.00646	0.3876
0.5	0.02508	1.5048
0.3	0.04700	2.8200

Supplementary Table 6: Calculated water volume slope for current and potential hold conditions.

Current density [mA cm^{-2}]	Water slope [mm^3 s^{-1}]	Water slope [mm^3 min^{-1}]
100	0.00938	0.5625
200	0.01875	1.1250
300	0.02813	1.6875
400	0.03750	2.2500
500	0.04688	2.8125
600	0.05625	3.3750
700	0.06563	3.9375
**Potential [V]**	**Water slope [mm^3 s^{-1}]**	**Water slope [mm^3 min^{-1}]**
0.7	0.02017	1.2102
0.5	0.04297	2.5782
0.3	0.07239	4.3434

Fitting function:

$$f(t) = mt + y$$

100 mA/cm^2: 0.1938 mm^3/min

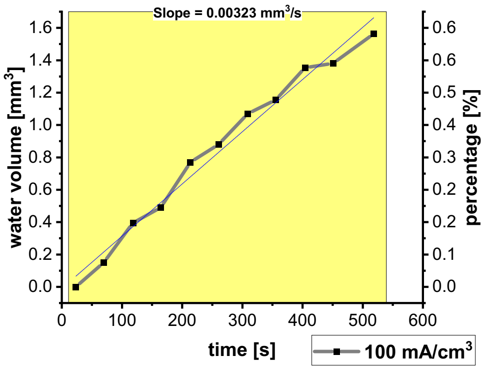

200 mA/cm^2: 0.4788 mm^3/min

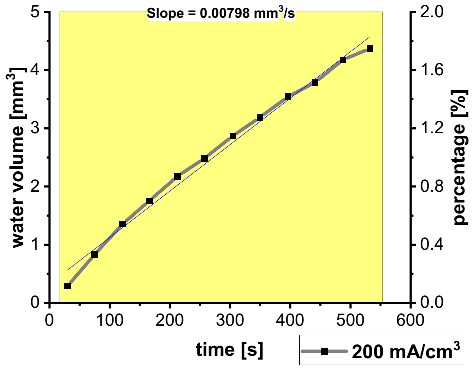

300 mA/cm^2: 0.8436 mm^3/min

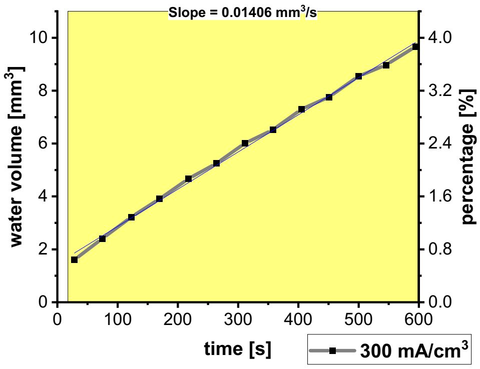

400 mA/cm^2: 1.3512 mm^3/min

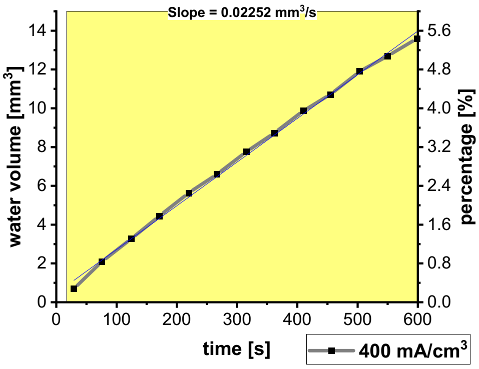

500 mA/cm^2: 1.8954 mm^3/min

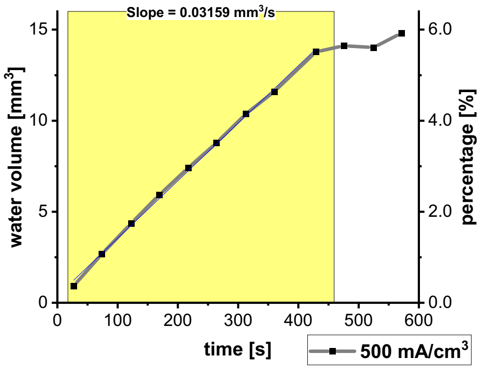

600 mA/cm^2: 2.4522 mm^3/min

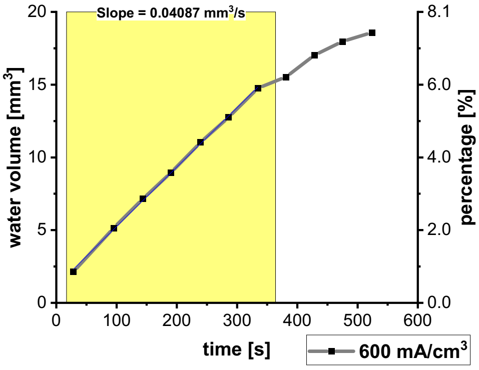

700 mA/cm²: 2.6850 mm³/min

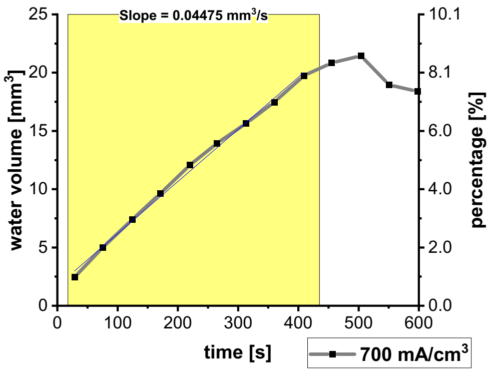

Supplementary Figure 19: Calculation of the water volume evolution inside the single serpentine PEFC for different current holds from 100 to 700 mA/cm².

0.7 V: 0.3876 mm³/min

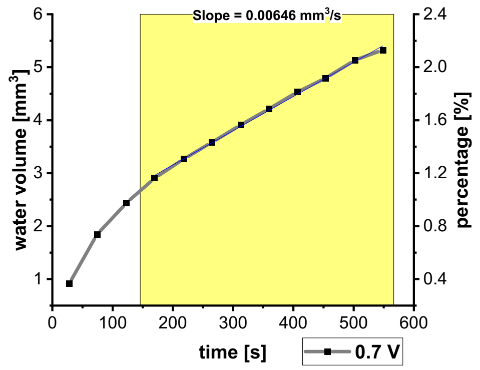

0.5 V: 1.5048 mm³/min

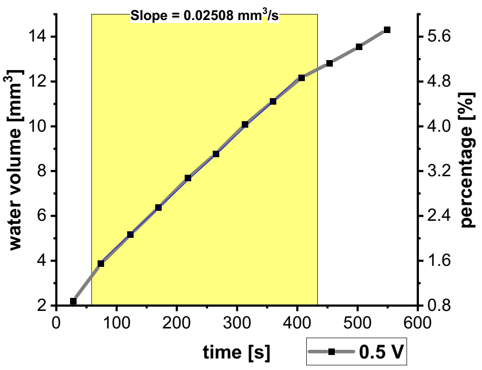

0.3 V: 2.8200 mm³/min

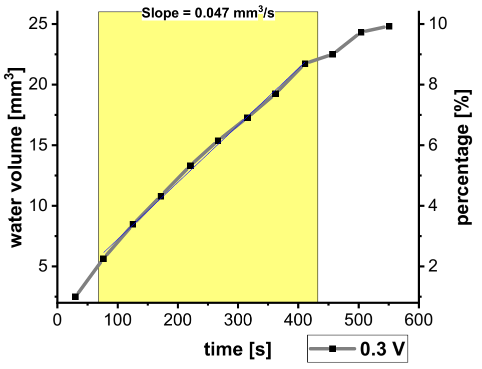

Supplementary Figure 20: Calculation of the water volume evolution inside the single serpentine PEFC for different potential holds from 0.7 to 0.3 V.
